# Quantitative angiographic radial artery diameter measurement and its relationship with common variables: A cross-sectional study

**DOI:** 10.1097/MD.0000000000043797

**Published:** 2025-08-08

**Authors:** Md Mahbub Hasan, Ashok Kumar Dutta, Md Rashid Al Mahmood, Smita Kanungo, Arifur Rahman, Md Rashidul Hassan

**Affiliations:** a Department of Cardiology, Ibrahim Cardiac Hospital and Research Institute, Dhaka, Bangladesh; b Department of Cardiology, National Heart Foundation Hospital and Research Institute, Dhaka, Bangladesh; c Department of Physical Medicine and Rehabilitation, Bangladesh Medical College, Dhaka, Bangladesh; d Department of Cardiology, National Institute of Traumatology and Orthopedic Rehabilitation (NITOR), Dhaka, Bangladesh.

**Keywords:** correlation, radial artery diameter, wrist circumference

## Abstract

Different techniques are available for peripheral artery diameter measurement. Angiographic assessment of the radial artery (RA) during coronary procedures offers a potentially cost effective, less hazardous and accurate method. Besides, it can provide some additional information. This cross-sectional study aims to calculate RA diameter (RAD) angiographically and to investigate its relationship with common parameters. A total of 230 patients undergoing percutaneous trans-radial coronary procedures at a tertiary cardiac hospital in Dhaka were included in the study to measure RAD using quantitative coronary angiography software. Patients’ height, weight, body mass index (BMI), and wrist circumference were recorded. The presence or absence of hypertension and diabetes was searched. The mean diameter of the RA was 2.35 ± 0.41 mm, and wrist circumference was 16.34 ± 1.05 cm. Males had significantly larger RADs (*P* < .001). No significant relation was encountered between RAD and hypertension (HTN) and diabetes mellitus. There was no significant correlation of RAD with BMI (*r* = −0.028, *P* = .676). However, wrist circumference was found to be positively associated with RAD (*R* = 0.214, *P* = .001). RAD although had a positive correlation with wrist circumference, but there was no relationship with HTN, diabetes mellitus, and BMI.

## 1. Introduction

Ischemic heart disease is one of the major public health problems in developed countries and is on the rise in developing countries.^[1]^ Percutaneous coronary interventions (PCI) are one of the effective management techniques for ischemic heart disease, which are done through either trans-femoral, trans-radial, trans-ulnar, distal radial or trans-brachial route. Although trans femoral is most common, trans-radial interventions (TRI) are gaining popularity in recent years.

Trans-radial approach favors better outcomes than trans-femoral regarding bleeding and vascular complications, especially in patients with acute coronary syndrome.^[2]^ Growing evidence, including American College of Cardiology, American Heart Association, the Society for Cardiovascular Angiography and Interventions, and European Society of Cardiology guidelines, supports the adoption of trans-radial route to improve acute coronary syndrome-related outcomes (radial first strategy).^[3–5]^ TRI does not need prolonged immobilization. This comparatively cost-effective procedure is convenient for female patients considering religious values and social customs of the Southeast Asian region. Overall, this approach seems to be better with respect to patients’ satisfaction and comfort.^[6]^

TRI has a higher rate of procedural failure because of its smaller size and high incidence of spasm in the radial artery (RA). The Inner lumen of the RA is a risk factor for RA injury and subsequent RA occlusion.^[7–9]^

In general, the RA diameter (RAD) of the western population (3.6 ± 0.8) is larger than the Asian (2.63 ± 0.35). RAD is associated with some anthropometric parameters like body mass index (BMI), wrist circumference, height, etc, and this association may help the interventionist to predict the size of instrument before coronary intervention.^[10–12]^

Furthermore, the presence of several clinical variables like hypertension (HTN), diabetes mellitus (DM) and gender may influence the size of the RA.^[11]^

There is little data on RAD and its influencing factors among the Bangladeshi population. One study showed RAD of the Bangladeshi population is positively correlated with BMI,^[13]^ but in other studies, which were done in India and Pakistan, they found no such correlation.^[11,14]^ Furthermore, no studies have measured angiographic post-vasodilated RAD in Bangladeshi population.

To search the mean diameter of RA in Bangladeshi population and to find the relationship between RAD and BMI, wrist circumference, HTN and DM were the objectives of the present study.

## 2. Materials and methods

The present cross-sectional study was conducted in a tertiary hospital of Bangladesh during the year 2022 (January–December). Both male and female adult (age >18 years) patients undergoing percutaneous coronary intervention through trans-radial approach for the first time during the study period were included in the study. Those having Radial and ulnar artery malformation, radial and ulnar artery Vaso-occlusive disease, recent radial angiography within past 3 months, local infection around puncture site, previous history radial graft, severe anemia, allergy to radio iodine contrast, severe comorbidity (e.g.: end stage renal disease) or unwillingness to participate were excluded from study

## 3. Data collection techniques

A total of 230 participants were included as per the inclusion and exclusion criteria. Informed consent was taken from each subject before enrollment. All demographic data, detailed history and baseline clinical data were recorded. Necessary investigations before the procedure were done as per hospital protocol. Body weight and height were measured using standard methods and devices. Wrist circumference was measured with the help of a measuring tape in centimeters at the level of the patient’s wrist line. Each patient was evaluated for major cardiovascular risk factors, including HTN and DM. The procedure room was air conditioned and temperature was kept between 20° and 25°.

### 3.1. Radial arteriography procedure

The trans-radial procedure was performed with analgesia and conscious sedation with either 25 mg pethidine or 3 mg morphine. Before the arterial puncture, local anesthesia was administered with 2 to 3 mL of 2% lidocaine. RA puncture was made with a 19-gauge needle at an angle of 25° to 30°, 1 to 2 cm proximal to the styloid process or 2 to 3 cm proximal to the wrist line. Upon peri-procedural bleeding, a guide wire was inserted through the needle. Then the 6Fr Terumo RadioFocus introducer II sheath and the dilator in it were introduced after a small percutaneous incision. Following the removal of the dilator and guide wire, a mixture consisting of 200 µg nitroglycerin, 1.25 mg verapamil, and 2500 IU heparin prepared in 10 mL of saline was administered. Radial arteriography was performed using contrast media diluted in saline before the coronary procedure (angiogram and angioplasty). Longer duration of procedure or post-procedural arterial spasm may result in variable diameter result; so, we took measurements at beginning in all participants.

The patient’s RAD was measured proximal to the vascular access sheath using a specially designed measurement program, quantitative angiography (QA), which took the radial vascular access sheath diameter as a reference. We had selected a healthy arterial segment proximal to the vascular access sheath, and computer generated minimal internal diameter was recorded as RAD. A photograph of the measurement was taken, and a printed copy was attached to the data collection form.

Hemostatic control was accomplished with the use of appropriately-sized sponge rolls after the procedure, and was followed up for complications according to hospital protocol.

All necessary information was recorded in predesigned, structured case record forms, and finally, all the relevant data were processed and presented in a tabulated format.

### 3.2. Statistical analysis

Statistical analysis of the data was performed using the IBM Statistical Package for Social Science version 26 (SPSS 26) statistical software (IBM Corporation, Armonk). While continuous variables were presented as mean ± standard deviation, categorical variables were presented as numbers of cases (n) and corresponding percentages (%). Continuous variables were compared between groups using Student *t*-test. Correlations between continuous variables were evaluated by Pearson correlation coefficient. The level of significance was set at 5% and a *P*-value of <.05 was considered statistically significant.

### 3.3. Ethical implication

Before commencement of the study, the research protocol was ethically reviewed and approved by the Ethical Review Committee (reference- N.H.F.H and R.I 14-14/7/Ad/974, date of approval-21/04/2022). Consent was taken from each patient after informing them of the objectives of the study, the risks and benefits, the confidential handling of personal information, the voluntary nature of participation and the rights to withdraw from study. Detailed study related information was read out and explained in the printed handout. The study conforms to the code of ethics of the World Medical Association (Helsinki Declaration).

## 4. Results

Table 1 shows mean age of the study subjects was 52.15 ± 9.99 years. 54.8% patients were hypertensive, while 30% were diabetic, and 5.2% were smokers. Mean BMI was 26.10 ± 4.02 Kg/m^2^, with 45.7% having normal body weight. Mean wrist circumference was 16.34 ± 1.05 cm. Table 2 shows the distribution of RAD according to different size categories. The mean diameter of the RA was 2.35 ± 0.41 mm. 15.7 % study subjects had <2 mm, 29.1% had more than 2.5 mm, and 55.2% had between 2.0 and 2.5 mm diameter. Hypertensive patients had larger diameter (2.38 ± 0.42mm) in comparison to non-hypertensive (2.30 ± 0.41mm) but this difference was not significant (*P* = .133). No significant difference was found between RAD of diabetic (2.35 ± 0.39 mm) and nondiabetic (2.34 ± 0.42 mm) subjects (*P* = .895). Diameter of RA was significantly greater in male subjects in comparison with their female counterpart (2.49 ± 0.39 mm; vs 2.19 ± 0.38 mm; *P* < .001) (Table 3). Table 4 shows the correlation of RAD with BMI and wrist circumference. There was a significant weak positive correlation of RAD with wrist circumference, but there was no significant correlation of RAD with BMI.

**Table 1:** Distribution of risk factors of the study subjects (N = 230).

Risk factors	Frequency (n)	Percentage (%)
Smoking		
Male	12	5.2
Female	0	0.0
Hypertension	126	54.8
Diabetes mellitus	69	30.0
BMI (kg/m^2^)		
Under weight (<18.5)	2	0.9
Normal weight (18.5–24.9)	105	45.7
Over weight (25.0–29.9)	88	38.3
Moderately obese (30.0–34.9)	24	10.4
Severely obese (35.0–39.9)	11	4.8

Mean BMI: 26.10 ± 4.02 (kg/m^2^). Mean age: 52.15 ± 9.99 years. Mean wrist circumference: 16.34 ± 1.05 cm.

BMI = body mass index.

**Table 2:** Radial artery diameter of the study subjects (N = 230).

RAD (mm)	Frequency (n)	Percentage (%)
<2.00	36	15.7
2.00–2.50	127	55.2
>2.50	67	29.1

Mean: 2.35 ± 0.41 mm.

RAD = radial artery diameter.

**Table 3:** Relation between RAD and hypertension, diabetes mellitus, gender (N = 230).

RAD*	Mean ± SD mm	*P*-value
Hypertension		
Yes	2.38 ± 0.42	.133
No	2.30 ± 0.41	
Diabetes mellitus		
Yes	2.35 ± 0.39	.895
No	2.34 ± 0.42	
Gender		
Male	2.49 ± 0.39	<.001
Female	2.19 ± 0.38	

RAD = radial artery diameter, SD = standard deviation.

* Data were analyzed using unpaired *t*-test and were presented as mean ± SD mm.

**Table 4:** Correlation of RAD with BMI, wrist circumference (N = 230).

RAD	*r*	*P*-value
BMI	−0.028	.676*
Wrist circumference	0.214	.001*

BMI = body mass index, RAD = radial artery diameter.

* Pearson correlation was done.

## 5. Discussion

This was the first study in Bangladesh that measured the RAD angiographically, and some factors were also evaluated to find out the determinants of radial artery (RA) size. The mean age of the patient was 52.15 years (range 28–87years), which was similar to other studies.^[13,15]^

The RAD was 2.49 ± 0.39 mm for men, 2.19 ± 0.38 mm for women and 2.35 ± 0.41 mm overall. One study,^[13]^ which was done in 2013, revealed the mean RAD of the Bangladeshi population 2.2 ± 0.3 mm. However, they used ultrasonography as a measuring tool while we used angiography in the present study.

A study was done in southern Rajasthan in, 2011, which revealed the mean RAD 2.325 ± 0.4 mm.^[10]^ Another recent large volume multicenter study^[11]^ in India found RA diameter median 2.8 mm, interquartile range (IQR): 2.4 to 3.1 mm. Loh et al^[16]^ found the mean right RAD 2.45 ± 0.54 mm in the Singaporean and Yan et al^[17]^ found the mean RA diameter 2.38 ± 0.56 mm in the Chinese population. In Pakistan, Ashraf et al^[14]^ found RAD 2.3 ± 0.4 mm in their local population. Other studies described the mean RAD of Korean^[18]^ 2.63 ± 0.35 mm, and for the Japanese, the diameter was 3.10 ± 0.60 mm in male patients and 2.80 ± 0.60 mm in female patients^[19]^

The RA mean diameter in this study is smaller than the Western population. Similarly, the mean diameter was larger in the Korean^[18]^ and the Japanese^[19]^ populations. But the findings of this current study are similar to other countries of this subcontinent like India,^[10]^ Pakistan.^[14]^

Considering the above findings, the RA diameter has some racial variation, but our finding is similar to other South East Asian population, and it is not unexpected. Moreover, techniques of measurement are an important issue, and different techniques were adopted in the above mention studies; most were ultrasonographical measurements except the Turkish^[15]^ and ours one, which were angiographical measurements. The mean RA diameter of our study is a bit larger than the previous study by Chowdhury et al^[13]^ They took measurements by ultrasound.

Many studies^[10,11,13,14,16]^ have found larger RA diameters in males than the females, which has been correlated well with the current study.

This study reveals no significant variation of RA diameter with regards to HTN, and this finding is similar to that of Dharma et al,^[11]^ Chowdhury et al,^[13]^ Yan et al.^[17]^ In contrary Beniwal et al,^[10]^ Loh et al,^[16]^ Khder et al,^[20]^ and Ashraf et al^[14]^ found a larger diameter in the hypertensive population.

The present study revealed no significant influence of DM on RA diameter and it is similar to findings of other investigators.^[10,13,14,19]^

The current study found no significant correlation of RA diameter with BMI, similar to Beniwal et al,^[10]^ Ashraf et al,^[14]^ and Saito et al,^[19]^ including the large volume multicentered study by Dharma et al.^[11]^

We found a weak positive correlation of RA diameter with wrist circumference (*R* = 0.214, *P* = .001). Okuyan et al,^[15]^ Aykan et al,^[21]^ Goswami et al^[22]^ found similar finding.

Angiography, a cornerstone in cardiovascular imaging, has proven indispensable in diagnosing and treating cardiovascular disorders. Its role extends beyond mere visualization, serving as a diagnostic compass that aids in formulating effective treatment strategies.^[23]^

Quantitative coronary angiography is valid and reliable in measuring arterial diameter. It provides more accurate and reproducible measurements than visual estimation. Geometric information, such as minimal luminal diameter, reference diameter, and percent diameter stenosis, represents the most useful and reliable information obtained by this system. This software can be used for QA of the peripheral vessels also.^[23,24]^ We targeted the RA, as nowadays it is commonly used for coronary interventions.

Ultrasound (USG), IVUS (intra vascular ultrasound) need more expenditure and a different set-up. Not all cardiac lab has facilities for IVUS. In contrast, QA emerges as a relatively cost-effective method; it can provide more accurate results. There is less chance for interobserver and intraobserver measurement differences in comparison to other techniques. QA is also better than angioscopy to some extent.^[23–26]^

RA occlusion occurs in 1% to 10% of patients undergoing trans-radial catheterization. In fact, more than 50% of trans-radial operators usually don’t assess RA patency routinely before hospital discharge. About 67% of radial arteries had intimal tears and 36% had medial dissections immediately after trans-radial PCI. Minimal lumen diameter and minimal lumen area are smaller in repeat trans-radial patients than in first-timers.^[27]^ Quantitative computerized measures of vessel correlate with several known predictors of large vessel peripheral vascular disease.^[26]^ Quantitative angiographic mean RAD measurement can give some direct evidence of arterial condition, and assessing interrelations to common predictors can provide some important information which can be utilized further for more observational and comparison studies. So, we had an endeavor to measure RAD among patients undergoing coronary procedures by QA, without any additional expenditure or setting like USG and search for some relationships with common variables.

## 6. Limitations of the study

This study has the following limitations:

1. It was conducted in a single center, so the study population may not represent the whole community.
2. There are many variables that may have an impact on RAD. We considered some of them in the present study; other variables can be studied in the future.
3. The sample size was relatively small.
4. We measured the RAD of only 1 hand.
5. Measurement of blood pressure and grading of hypertension were not taken into consideration.

## 7. Recommendation

The following recommendations are proposed for further studies:

1. Further studies with a large sample size will be helpful to reach a consensus.
2. A multicenter study can be carried out.
3. Comparison can be done between ultrasonography guided and angiographically measured RAD.
4. Routinely, RA assessment by QA can be done among patients undergoing complex PCI to get real-time information on the RA.

## 8. Conclusion

This study revealed angiographically measured average diameter of the RA was 2.35 ± 0.41 mm. Although we found a weak positive correlation between RAD and wrist circumference, there was no relationship with HTN, DM and BMI.

Supplemental digital contents “Appendix and Figure” are available for this article (https://links.lww.com/MD/P607).

## Author contributions

**Conceptualization:** Md Mahbub Hasan, Ashok Kumar Dutta, Md Rashid Al Mahmood, Smita Kanungo, Arifur Rahman, Md Rashidul Hassan.

**Data curation:** Md Mahbub Hasan.

**Investigation:** Md Mahbub Hasan.

**Methodology:** Md Mahbub Hasan.

**Supervision:** Ashok Kumar Dutta.

**Writing –original draft:** Md Mahbub Hasan, Md Rashid Al Mahmood.

**Writing – review & editing:** Md Mahbub Hasan, Md Rashid Al Mahmood.

## Supplementary Material


