# Supplementary material for: Quantitative angiographic radial artery diameter measurement and its relationship with common variables: A cross-sectional study
Source: Medicine (Baltimore). 2025 Aug 8;104(32):e43797. doi: 10.1097/MD.0000000000043797 (PMC12338288; doi:10.1097/MD.0000000000043797)
Supplement: Supplementary file 1 [file medi-104-e43797-s001.docx]

**Appendix**

**Quantitative coronary angiography (QCA) :** QCA is now the "gold standard" for the assessment of the coronary tree. The important parameters which can be obtained by edge detection are MLD (minimal luminal diameter), maximal luminal diameter, mean luminal diameter, lesion length. The principles of QCA, which were initially designed for diagnostic studies of coronary artery disease (CAD), have necessarily been adapted to more complex situations related to either the presence of a device or the effect of an intervention on the angiographic appearance of a damaged vessel. ^*^

**Quantitative angiography (QA)** software can be used to measure peripheral arterial diameter.

*Reference-Foley DP, Escaned J, Strauss BH, di Mario C, Haase J, Keane D, Hermans WR, Rensing BJ, de Feyter PJ, Serruys PW. Quantitative coronary angiography (QCA) in interventional cardiology: clinical application of QCA measurements. *Prog Cardiovasc Dis.* 1994 Mar-Apr;36(5):363-84. doi: 10.1016/s0033-0620(05)80027-1.

^
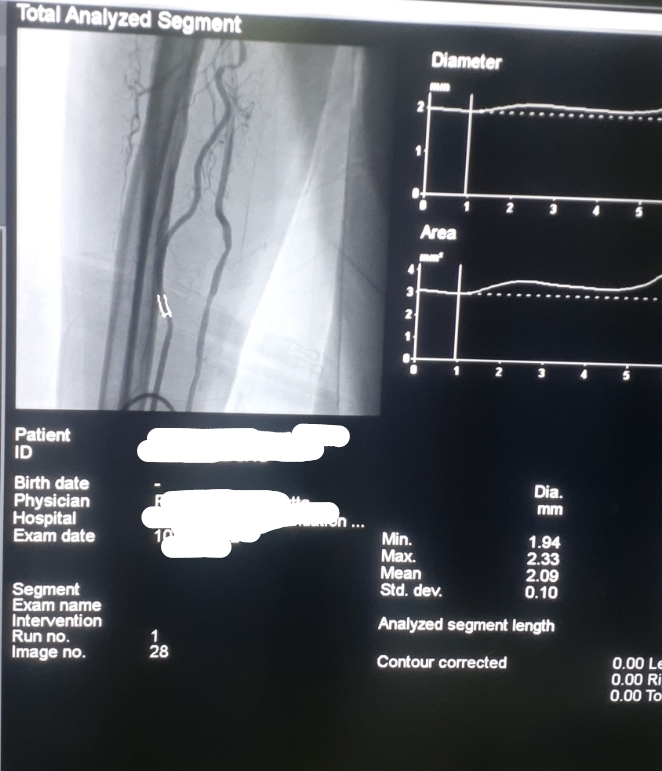
^

**Figure 1 -Right radial arteriography followed by measurements of radial artery diameter**
